# Population Genetic Structure of *Cnesterodon decemmaculatus* (Poeciliidae): A Freshwater Look at the Pampa Biome in Southern South America

**DOI:** 10.3389/fgene.2017.00214

**Published:** 2017-12-19

**Authors:** Aline M. C. Ramos-Fregonezi, Luiz R. Malabarba, Nelson J. R. Fagundes

**Affiliations:** ^1^Laboratory of Medical and Evolutionary Genetics, Department of Genetics, Federal University of Rio Grande do Sul, Porto Alegre, Brazil; ^2^Laboratory of Bioinformatics and Evolution, Department of General Biology, Federal University of Viçosa, Viçosa, Brazil; ^3^Laboratory of Ichthyology, Department of Zoology, Federal University of Rio Grande do Sul, Porto Alegre, Brazil

**Keywords:** Bayesian phylogeography, mitochondrial DNA, neotropical ichthyology, Pampa biome, stream capture

## Abstract

The Pampas is a Neotropical biome formed primarily by low altitude grasslands and encompasses the southernmost portion of Brazil, Uruguay, and part of Argentina. Despite the high level of endemism, and its significant environmental heterogeneity, Pampean species are underrepresented in phylogeographic studies, especially aquatic organisms. The Pampean hydrological system resulted from a long history of tectonism, climate, and sea level changes since the Neogene. In this study, we examined the population genetic structure of *Cnesterodon decemmaculatus*, a freshwater fish species that occurs throughout most of the Pampa biome. We characterized mitochondrial and autosomal genetic lineages in populations sampled from Southern Brazil and Uruguay to investigate (1) the correspondence between current drainage systems and evolutionary lineages, (2) the demographic history for each genetic lineage, and (3) the temporal depth of these lineages. Overall, we found that the major evolutionary lineages in this species are strongly related to the main Pampean drainage systems, even though stream capture events may have affected the distribution of genetic lineages among drainages. There was evidence for recent population growth in the lineages occupying drainages closest to the shore, which may indicate the effect of quaternary sea-level changes. In general, divergence time estimates among evolutionary lineages were shallow, ranging from 20,000 to 800,000 years before present, indicating a geologically recent history for this group, as previously reported in other Pampean species. A Bayesian phylogeographical reconstruction suggested that an ancestral lineage probably colonized the Uruguay River Basin, and then expanded throughout the Pampas. This evolutionary scenario may represent useful starting models for other freshwater species having a similar distribution.

## Introduction

The South American Pampa, or “Pampas,” is a Neotropical biome dominated by natural grasslands spreading over plains of Uruguay, Northern Argentina, Southern Brazil, and part of Paraguay (Pallarés et al., 2005). However, from the biological standpoint, the Pampas are far from homogeneous. Indeed, many proposals for small biological “provinces” have been made, especially regarding on the different plant communities (Cabrera and Willink, 1980; Overbeck et al., 2007), which reflects the mosaic of soil types resulted from its complex geological history. At a smaller scale, such heterogeneity leads to high species endemism and significant genetic structure in Pampean species studied so far (e.g., Freitas et al., 2012; Turchetto et al., 2014; Felappi et al., 2015). While some phylogeographic studies in the Pampas have found little geographic structure and shallow gene trees [<0.1 million years ago (mya)] (Speranza et al., 2007; Turchetto et al., 2014), other studies found strong geographic structure and a deep mitochondrial gene tree (>2.5 mya) (Felappi et al., 2015). However, these studies did not include freshwater organisms, and, therefore, it is difficult to predict the level and depth of the genetic structure exhibited by these species. Species occurring in the Pampa biome have been underrepresented in studies of phylogeography and conservation genetics (Lawler et al., 2006; Beheregaray, 2008; Turchetto-Zolet et al., 2013), which further complicates the understanding of general drivers of biological diversification in this biome.

The Pampas hydrological system resulted from a long history of tectonism, climate, and sea level changes since the Neogene (Casciotta et al., 1999). During the Quaternary, soil erosion and other marine regression/transgression cycles have continued to shape hydrological systems (Tonni and Cione, 1997; Quattrocchio et al., 2008) by altering the relationship among tributaries, isolated drainages, lagoons, and estuaries (Martin and Dominguez, 1994; Thomaz et al., 2015). These geomorphological processes promoted successive stream captures events, allowing obligate freshwater species to disperse using temporary connections (e.g., Loureiro et al., 2011; Candela et al., 2012; Grassi et al., 2017). Stream capture (also known as river capture, headwater capture, or drainage rearrangement) is a geomorphological process that consists in the contact between neighboring drainages. It occurs when the tributary of a river basin starts flowing toward a neighbor basin, and results in icthyofauna dispersal between them (Bishop, 1995). Temporary connections between adjacent basins caused by fluctuations in the sea level during glacial–interglacial cycles may have also influenced the current distribution of several freshwater fish genera (e.g., Ketmaier et al., 2004; Swartz et al., 2007, 2009; Thomaz et al., 2015), including the Pampean taxa *Australoheros*, *Cnesterodon*, *Jenynsia*, and *Corydoras* (e.g., Bruno et al., 2011, 2016; Ponce et al., 2011).

It is well accepted that the distribution of freshwater fish lineages mainly reflects the paleogeography of a specific region (Bermingham and Avise, 1986; Bernatchez and Wilson, 1998; Avise, 2000; Lévêque et al., 2008). *Cnesterodon decemmaculatus* (Jenyns 1842) is endemic to the Pampa biome and is one of the most widespread freshwater fish species in this region. It is found in the freshwater Ecoregions 332 (lower Uruguay) and 334 (laguna dos Patos basin) described by Abell et al. (2008), but exclusively in grassland environments in the southern part of these ecoregions, associated to the Pampa biome, being absent in the northern portion of these ecoregions located in the Atlantic Forest Biome. It is further found in the freshwater ecoregions 345 (Subtropical Potamic axis) and 347 (Bonaerensean Atlantic), also in the Pampas. Thus, this species constitutes an excellent model to examine historical processes that played a major role in shaping the genetic structure of freshwater fishes in the Pampas. In addition, this species can be found in a wide range of habitats, including rivers, ponds, and shallow wetlands, even though it has a poor swimming capacity in high-speed currents (Trenti et al., 1999). A recent study using *C. decemmaculatus* populations from the southern Pampean region showed low genetic divergence and evidences of recent colonization of this area (Bruno et al., 2016). However, this study focused on the coastal drainages from Argentina (ecoregion 347 – Bonaerensean Atlantic), which represent a small portion of the natural distribution of *C. decemmaculatus* in the Pampas.

In this study, we used mitochondrial and nuclear genetic markers to evaluate the genetic structure of *C. decemmaculatus* over its distribution. More specifically, we were interested in answer the following questions: (1) How strong is the correspondence between current drainage systems and evolutionary lineages? (2) Is there evidence of ancient population growth or reduction for each genetic lineage in this species? (3) What is the temporal depth for the evolutionary divergence among lineages and populations? (4) What is the colonization history for this taxon in the Pampas?

## Materials and Methods

**FIGURE 1:**
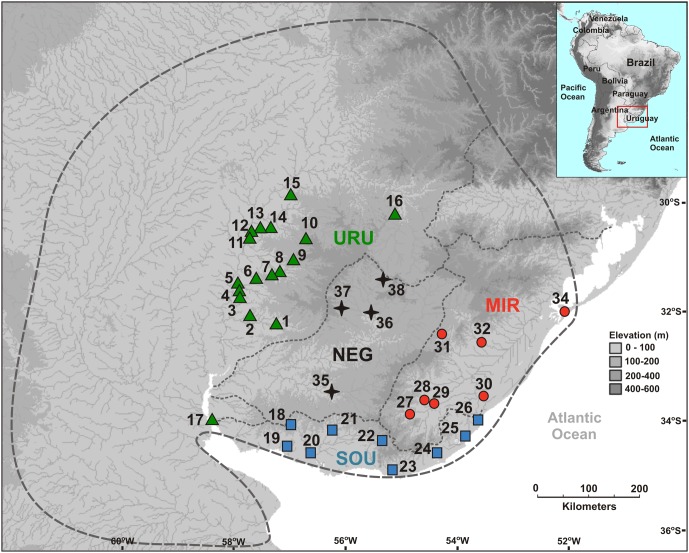
Geographical location of sampled sites showing the limits of the major drainage systems (assumed as different populations). The dashed gray line indicates the distribution of *Cnesterodon decemmaculatus* according to Lucinda, 2005. Vouchers of the specimens used in the study, and the geographical coordinates for sampling sites are available in Supplementary Table S1.

We sampled 99 individuals from 37 localities (**Figure 1**), covering a significant part of the species’ range. Our sampling ranged from one to eight individuals per locality (Supplementary Table S1). All individuals included in our analyses were fixed in 96% ethanol and deposited in the fish collection at the Federal University of Rio Grande do Sul (Universidade Federal do Rio Grande do Sul – UFRGS). All collections were performed under the approval of the government authorities of Brazil and Uruguay (Ministério do Meio Ambiente, Brazil – SISBIO, license number 12038-2, and Dirección de Recursos Naturales del Ministerio de Ganadería, Agricultura y Pesca, Uruguay). The collection and euthanasia of the specimens were approved by the ethics committee of the UFRGS, license number 24434.

Total genomic DNA was isolated from muscle tissue with cetyltrimethyl ammonium bromide (CTAB) as described by Doyle and Doyle (1987). We tested two mitochondrial (mtDNA) genes: cytochrome oxidase subunit I (*COI*) (Herbert et al., 2003) and NADH dehydrogenase 2 (*ND2*) (Sorenson, 2003) and two nuclear (nDNA) genes: SH3 and PX domain containing 3-like protein (*SH3PX3*) and myosin heavy chain 6 (*Myh6*) (Li et al., 2007). However, *SH3PX3* and *COI* had insufficient variation in a preliminary sample of 10 individuals per basin and, therefore, were excluded from further analysis. PCR amplification protocols for *ND2* and *Myh6* followed Sorenson (2003) and Li et al. (2007), respectively. PCR products were checked on a 1% agarose gel, purified with Exonuclease I and Shrimp Alkaline Phosphatase (GE Healthcare^®^) and sequenced using the Sanger method at Macrogen Inc. (Seoul, South Korea). Both DNA strands were sequenced, checked, and aligned with GeneiousPro v.4.8 (Drummond et al., 2007). Haplotypes of the *Myh6* gene were estimated in PHASE 2.1 (Stephens et al., 2001) using 10,000 steps sampling every 10 steps and discarding the first 1000 steps as burnin. We ran PHASE several times using different starting seeds to ensure the reliability of the final estimate. Mitochondrial haplotypes were defined using DnaSP 5.10 (Rozas et al., 2003). All sequences obtained in the present study were deposited in GenBank (KU214332–KU214430 and KU214252–KU214331, for *ND2* and *Myh6* genes, respectively).

Dispersal of obligatory freshwater fish requires connections of aquatic habitats between adjacent basins and, most of taxa have their distribution range narrowly coincident, if not equal, with the hydrographic basins boundaries in which they live (Ward et al., 1994; Albert and Reis, 2011). Based on this, and also because we found a general genetic structure associated to major Pampean drainages, we defined four geographical groups for population analysis: Uruguay River Basin (URU), Negro River Basin (NEG), Mirim Lagoon Basin (MIR), and Southern coastal Basins of Uruguay (SOU) (**Figure 1** and Supplementary Table S1). Because independent drainage systems represent plausible boundaries for isolated biological populations for freshwater organisms, individuals collected in different localities within each basin were merged into a single population, resulting in a sample of 62 individuals for URU, 11 for NEG, 15 for MIR, and 11 for SOU. Following Abell et al. (2008), URU and NEG represent the freshwater Ecoregion 332 (lower Uruguay), MIR represents Ecoregion 334 (laguna dos Patos), while SOU have populations located in both Ecoregions 334 and 345 (lower Paraná).

To determine the correspondence between current drainage systems and evolutionary lineages, we inferred the evolutionary relationship among haplotypes using a median-joining network (Bandelt et al., 1999) estimated in NETWORK 4.1.0.9^1^. The resulting genetic structure was quantified using an analysis of molecular variance (AMOVA) (Excoffier et al., 1992) and pairwise Φ*_ST_*s calculated in Arlequin 3.5 (Excoffier and Lischer, 2010). In addition, we tested for population structure without assuming any hypothesis based on hydrography using the Bayesian clustering algorithm implemented in BAPS (Corander et al., 2008). We used the mixture population model and set the maximum number of populations (*K*) to 37, the number of sampled local populations. Because stream capture events may affect gene genealogies and molecular diversity patterns within drainages, we decided to run several analyses excluding localities for which we may have evidence for stream capture. We inferred a possible stream capture event whenever we found shared haplotypes between neighbor drainages, especially when populations closely related to watersheds were involved (see the section “Discussion” for further details for each case).

To test for evidence of ancient population growth or reduction for each population (URU, NEG, MIR, and SOU) we first estimated descriptive statistics of genetic diversity, such as nucleotide (π) and haplotype diversity (*H*), followed by Tajima’s *D* (Tajima, 1983) and Fu’s *F*_S_ (Fu, 1997) neutrality tests, which were calculated in Arlequin 3.5 (Excoffier and Lischer, 2010). We performed neutrality tests under two schemes: considering all individuals for a given basin, and excluding localities for which there was evidence of headwater capture. We also estimated the effective population size (*N*_E_) and the population growth parameter (*G*) for each population without the localities with evidence for headwater capture (namely URU 7, URU 8, NEG 35, NEG 36, MIR 31), using both markers in the program LAMARC v. 2.1.6 (Kuhner, 2006). We performed a maximum-likelihood search based on three replicates of 10 initial chains and two final chains. The initial chains consisted of 250 samples drawn every 20 steps, and a burn-in of 1000 samples for each chain. The two final chains used the same burn-in and sampling interval, but consisted of 10,000 samples. We assumed a generation time of 1 year based on estimates of sexual maturity for the poeciliid *Poecilia reticulata* (Reznick and Bryga, 1987).

Finally, we inferred the divergence times among populations and the colonization history for *C. decemmaculatus* based on two approaches. First, we estimated a time-calibrated gene-tree genealogy for mtDNA lineages using Bayesian inference in BEAST 1.7.5 (Drummond and Rambaut, 2007). We used a coalescent constant-size tree prior with a random starting tree and the TN93+G model of sequences evolution, as determined by the corrected Akaike Information Criterion (AICc) in jModelTest2 (Darriba et al., 2012). We used each drainage as a discrete trait to allow the estimation of the most likely location of all ancestors in the mtDNA tree. While this may be indicative of dispersal events, it should not be viewed as a formal test of stream capture hypotheses. The MCMC was run twice for 10 million generations each, and 10% of the samples was discarded as burn-in. A normal strict molecular clock was assumed, calibrated with a normal distribution for molecular substitution rate of 8.6 ± 0.1 × 10^-9^ substitutions per site per year (s/s/y), which has been suggested in the literature for Cyprinodontiformes based on a dataset which included the *ND2* gene (Hrbek and Meyer, 2003).

Second, we used a coalescent-based species-tree analysis (Heled and Drummond, 2010) to infer the colonization history of each river basin using the StarBeast model in the program BEAST 1.7.5 (Drummond and Rambaut, 2007). For this analysis, we used populations as terminals, and used both markers with unlinked gene-trees (mitochondrial – *ND2* and nuclear – *Myh6*). We excluded localities showing evidence for headwater capture, as the species-tree method does not allow gene flow among terminals. We used a rate-reference prior to calibrate the molecular clock for *Myh6* (Ferreira and Suchard, 2008). Under this approach, a time-calibrated genealogy (i. e., *ND2*) is taken as a reference to estimate the relative molecular clock associated with alternative gene trees (i.e., *Myh6*). This is a useful approach when different partitions evolve at different rates, but there is no prior information on the molecular clock for all independent partitions in the matrix (Ferreira and Suchard, 2008). All remaining parameters and priors followed the Bayesian phylogenetic analyses mentioned above. In all Bayesian analysis, sampling sufficiency was evaluated by monitoring effective sample size (ESS) and ensuring that all values were higher than 200.

## Results

**FIGURE 2:**
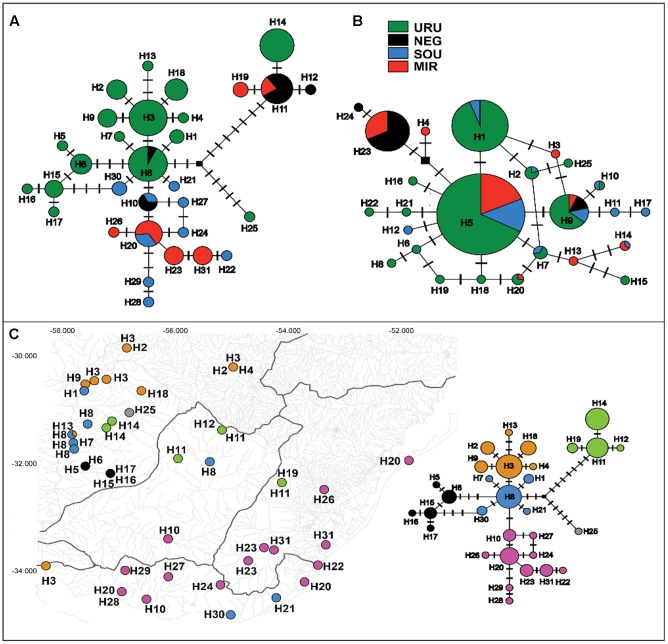
Median-joining networks for **(A)** mtDNA *ND2* haplotypes, and **(B)** nDNA *Myh6* haplotypes. Circles size are proportional to the observed frequency of each haplotype. Cross marks represent mutational differences between haplotypes. **(C)** Geographic location of each mtDNA haplotype. Different colors represent the six genetic groups identified in BAPS analysis.

Ninety-nine sequences were obtained for the mtDNA *ND2* gene (980 bp) yielding 31 different haplotypes defined by 42 variable sites (28 parsimoniously informative). For the autosomal *Myh6* gene we had 188 sequences (681 bp) resulting in 25 different haplotypes defined by 21 variable sites (16 parsimoniously informative). In general, the mtDNA haplotype network showed little haplotype sharing among populations, and a close relationship among haplotypes from the same population (**Figure 2A**). Populations of MIR and SOU exhibited related haplotypes, except for two SOU localities that had haplotypes H21 and H30, which are more closely related to haplotypes found in URU. Concerning *Myh6* haplotypes (**Figure 2B**), URU, MIR, and SOU shared the most frequent haplotypes (H5 and H1), and H9 was the only haplotype present in populations of all drainage systems. The close relationship among haplotypes for both markers is suggestive of a shallow genealogical history for this species (see below).

Considering all samples, the Bayesian clustering analysis found *K* = 6 genetic populations (**Figure 2C**). There was a marked difference in the number of different genetic population occurring in each major drainage. Five genetic populations occurred in URU, three in NEG, and two in both SOU and MIR. Not considering samples possibly affected by stream capture events, we found four genetic populations in URU, two in SOU, and one in both NEG and MIR, suggesting significant genetic structure within URU mainly due to three genetic groups with a restricted geographical distribution in this drainage (**Figure 2C**).

**FIGURE 3:**
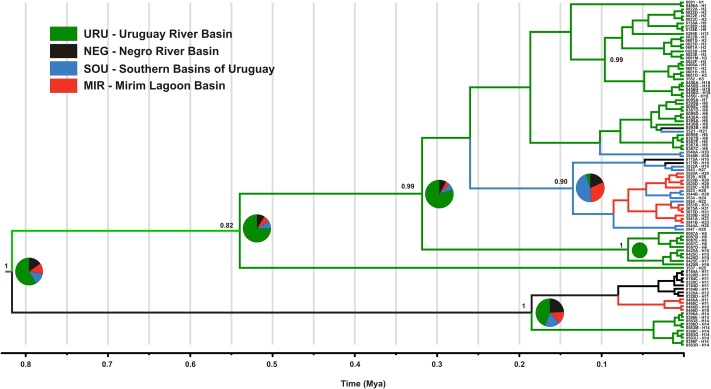
Maximum clade credibility tree among *ND2* lineages found in *Cnesterodon decemmaculatus* individuals. Posterior probabilities (*PP*) are shown above the branches for selected nodes with *PP* > 0.8. Pie charts beside selected ancestral node represent the *PP* of ancestral location among each of the four populations according to the color scheme shown in the inlet.

The mtDNA genealogy for *C. decemmaculatus* (**Figure 3**) suggests the most recent common ancestor (MRCA) of all mitochondrial lineages dates from 0.8 mya [95% credible interval (CI) 0.5–1.2 mya], and were most probably located in URU (*PP* = 0.58). A well-supported clade (posterior probability *PP* = 1.00), sister to all other lineages, occurred in isolated populations inhabiting elevated plains from three basins: URU, MIR, and NEG (haplotypes H11, H12, H14, and H19). These haplotypes represented one of the Bayesian genetic populations. However, the location of the MRCA for this clade was uncertain (*PP* < 0.5 for all locations). Its sister clade, which had moderate support (*PP* = 0.82) had URU as the most likely location (*PP* = 0.75), and showed H25 (another Bayesian genetic population) sister to a well-supported clade (*PP* = 0.99) that also has URU as its most likely location (*PP* = 0.80). In turn, this clade harbored four Bayesian genetic populations that corresponded to three well-supported clades (*PP* > 0.90) plus a “mixed” genetic population that included H1, H7, H8, H30, and H21. Regarding the most likely ancestral location, all remaining genetic groups had URU as the most likely location (*PP* > 0.85), with one exception. The clade, which was almost exclusive to MIR and SOU (though H10 also occurred in NEG), had SOU or MIR as its most likely ancestral location (*PP* = 0.46, *PP* = 0.30, respectively).

Considering the full dataset, both markers showed high haplotype diversities (*H* = 0.94 for *ND2* and *H* = 0.79 for *Myh6*). Both haplotype and nucleotide diversity were higher for URU and SOU compared with NEG and MIR (**Table 1**). There was significant genetic population structure (*ND2* Φ_ST_ = 0.26, and *Myh6* Φ_ST_ = 0.30, *P* < 0.001). Excluding localities that may represent stream capture events increased Φ_ST_ estimates (*ND2* Φ_ST_ = 0.59, and *Myh6* Φ_ST_ = 0.35, *P* < 0.001). For *ND2*, this was still lower than the Φ_ST_ estimate among the genetic populations delimited with BAPS (Φ_ST_ = 0.75, *P* < 0.001), which is expected, given that Bayesian populations were defined based on ND2 data. However, this trend was not followed by *Myh6* (Φ_ST_ = 0.19, *P* < 0.001). Pairwise Φ_ST_ values indicated that geographic groups were significantly different from each other except MIR vs. SOU (**Table 2**). Taking into account possible events of stream capture resulted in increased Φ_ST_ values between NEG and the remaining populations for both genetic markers, and between the other pairs only for *ND2* (**Table 2**). Because localities from URU might represent different genetic populations, we also quantified the genetic structure between them. For both *ND2* and *Myh6*, we found significant genetic structure in URU (0.81 and 0.39, respectively, *P* < 0.001), which was robust to the exclusion of some localities due to stream capture events (0.64 and 0.31, respectively, *P* < 0.001).

**Table 1:** Diversity indexes obtained for the mitochondrial and nuclear markers, respectively (*ND2* values/*Myh6* values), for the four populations considered in the study.

Population	*N*	*H*	π	Tajima’s *D*	Fu’s *F*_S_	Tajima’s *D*^‡^	Fu’s *F*_S_^‡^
URU	62/60	0.89/0.74	0.006/0.001	-0.48/-1.40	0.17/-9.36^∗∗^	-1.43/-1.49^∗^	-1.90/-10.17^∗∗^
NEG	11/9	0.60/0.46	0.004/0.001	0.78/-0.62	2.93/0.93	-1.05/0.15	-0.18/0.55
MIR	15/14	0.86/0.71	0.005/0.002	1.06/-0.29	2.27/-1.67	0.46/-0.71	-0.41/-1.94
SOU	11/11	0.96/0.79	0.003/0.002	-0.63/-1.13	-4.39^∗∗^/-5.24^∗∗^	–	–
All samples	99/94	0.94/0.79	0.007/0.002	-0.75/-1.46^∗^	-6.40/-16.76^∗∗^	–	–

*N, number of individuals sampled for ND2/Myh6 markers; *H*, haplotype diversity; π, nucleotide diversity; ^∗^P < 0.05; ^∗∗^P < 0.02; ^‡^excluding localities showing evidence of stream capture (see text).*

**Table 2:** Pairwise Φ_ST_ values for mtDNA *ND2* (lower diagonal) and nDNA *Myh6* (upper diagonal) markers.

Populations	URU	NEG	MIR	SOU
**All locations**				
URU	–	0.59	0.10	0.04
NEG	0.32	–	0.37	0.55
MIR	0.19	0.33	–	0.04^∗^
SOU	0.18	0.58	0.11	–
**Locations excl. SC^1^**				
URU	–	0.65	0.01^∗^	0.02^∗^
NEG	0.71	–	0.67	0.66
MIR	0.41	0.93	–	-0.02^∗^
SOU	0.33	0.88	0.14	–

*P < 0.05 for all values except when noted by an asterisk (^∗^). ^1^Excluding locations showing evidence of stream capture (see text).*

**FIGURE 4:**
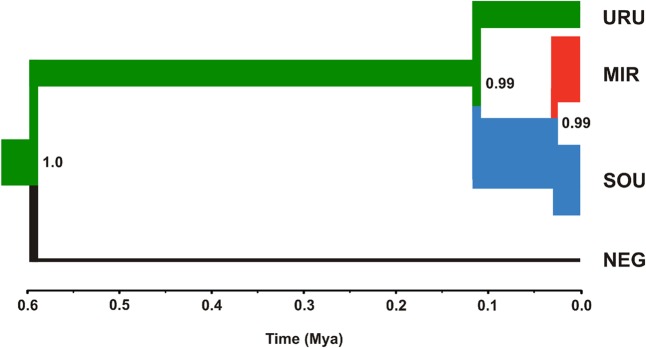
Maximum clade credibility tree considering *ND2* and *Myh6* under a coalescent approach. The thickness of the branches is proportional to effective population size (*N*_E_) estimates for each geographical group (see **Table 3** for more details). The color scheme for each branch followed the estimates of the most likely location for ancestral nodes, shown in **Figure 3**.

Neutrality tests provided mixed evidence for population growth in some populations. However, they were dependent on which statistic or genetic marker were used (**Table 1**). On the other hand, maximum likelihood-based demographic estimates revealed population growth for SOU and MIR (**Table 3**). Effective population size was also larger for SOU and MIR (∼3 × 10^6^ effective individuals), while NEG had the lowest value (∼180,000 effective individuals; **Table 3** and **Figure 4**). The combined analysis of mtDNA and nDNA markers suggested that the split between NEG and the clade URU+MIR+SOU, which was also the root of the tree, dates back from ∼0.6 mya (95% CI 0.27–0.95 mya). This was followed by the divergence between URU and MIR+SOU around 0.12 mya (95% CI 0.04–0.19 mya), and the subsequent divergence between MIR and SOU <30,000 years ago (95% CI 23,000–57,000) (**Figure 4**).

**Table 3:** Estimates for the effective population size (*N*_E_) and growth parameter (*G*) in all populations.

Population	Theta (𝜃) (95% CI)	*N*_E_ (95% CI)	*G* (95% CI)
URU	1.8 × 10^-2^	1.38 × 10^6^	–
	(1.5 × 10^-2^–2.1 × 10^-2^)	(1.18 × 10^6^–1.63 × 10^6^)	
NEG	2.4 × 10^-3^	1.82 × 10^5^	–
	(1.6 × 10^-3^–3.7 × 10^-3^)	(1.22 × 10^5^–2.86 × 10^5^)	
MIR	4.4 × 10^-2^	3.39 × 10^6^	2994.07
	(1.5 × 10^-2^–9.7 × 10^-2^)	(1.12 × 10^6^–7.44 × 10^6^)	(1314.72–5313.02)
SOU	4.7 × 10^-2^	3.62 × 10^6^	1015.58
	(2.3 × 10^-2^–10.1 × 10^-2^)	(1.78 × 10^6^–7.73 × 10^6^)	(393.40–1579.73)

*Theta (𝜃) = 4N_E_μ, where μ is the mutation rate per site per generation. 95% CI–95% credible interval for the estimates. There was no evidence for growth in URU and NEG (see the section “Materials and Methods”).*

## Discussion

In this study, we characterized the major genetic structure patterns of populations of *C. decemmaculatus* in most of its distribution in the Pampa biome. Differently from the star-like pattern found for Argentinean populations of *C. decemmaculatus* in the southern Pampas (Bruno et al., 2016), which indicates a very recent evolutionary history, our results reveal a significant genetic structure that, in general, paralleled broad drainage systems, especially considering mtDNA lineages (**Figures 2A**, **3**). However, there is no simple relationship between clades and basins, such that a single basin may contain more than one mtDNA clade, and different basins may share the same mtDNA haplotype (**Figures 2A**, **3**). Thus, understanding population structure imposed by the drainage system is necessary, but not sufficient, to account for the genetic patterns observed in our study. This is reinforced by the Bayesian clustering of populations, which suggested *K* = 6 as the bet number of genetic populations. Three alternative (but not exclusive) scenarios that may explain the discrepancy between drainage and genetic structure are: (1) migration among neighbor populations (active dispersal), (2) headwater capture (geodispersal), or (3) shared ancestral polymorphism. The first scenario seems unlikely because *C. decemmaculatus* has a poor swimming capacity in high-speed currents (Trenti et al., 1999), being unable to disperse actively across long distances. On the other hand, *Cnesterodon* species are commonly found in shallow wetland habitats (Maltchik et al., 2014), suggesting that geodispersal may represent a more likely alternative for these species. In this case, wetland habitats close to the watersheds between different drainages may facilitate headwater capture events, as minor environmental changes may reshape the hydrological network at these sites, impeding a clear-cut relationship between genetic lineages and drainages.

However, distinguishing between the geodispersal and ancestral polymorphism hypotheses may be more nuanced. This is further complicated by the fact that it is very difficult to set up an explicit test of stream capture hypotheses. One example of a likely case of stream capture involving closely related haplotypes occurring in different drainages is represented by mtDNA haplotypes H11, H14, and H19 (**Figure 3**). Even though URU has received higher support as the most probable location of the MRCA, mtDNA network analysis indicates that this clade evolved in NEG with subsequent stream capture events toward URU and MIR. First, there are seven exclusive substitutions in this clade (**Figure 2A**), suggesting it may have evolved in relative isolation. Second, the sampling points in URU and MIR containing these lineages are adjacent to NEG. Third, H14 and H19 are probably descendent from H11, which occurs in NEG (**Figure 2A**). Under this interpretation, this clade would reflect three independent stream capture events: NEG would have been colonized from URU between 0.2 and 0.8 mya (see below), while MIR and URU would have received migrants from NEG more recently (<0.2 mya). Even in the alternative scenario in which URU is the true location for the MRCA of this clade, two headwater capture events would have occurred, since this lineage would have reached NEG from URU by ∼0.2 mya, dispersing to MIR later on. Two other examples of haplotype sharing that may reflect stream capture toward NEG include H8 (from URU) and H10 (from SOU). In both cases, these haplotypes are more closely related to other lineages from specific drainages, and their sequence identity suggests a recent arrival in NEG. However, while H10 occurs in a site close to SOU, H8 occurs relatively distant from the watershed with URU. An alternative hypothesis for the occurrence of H8 in NEG would involve upstream active dispersal from lower URU populations despite the low dispersal ability of *Cnesterodon* (Trenti et al., 1999).

The tectonic activity associated with stream capture events in eastern South America may be as old as 1.6 mya (Saadi et al., 2002). The presence of H11 and H19 in MIR and H8 in NEG is in agreement with a pattern in which more eastern drainages capture upland shield drainages. However, the presence of H14 in URU and H10 in NEG indicates that these events could also occur in the opposite direction, suggesting a highly dynamic scenario in the Pampas, possibly because this biome is largely flat. Indeed, Loureiro et al. (2011) suggested drainage rearrangements in both directions across the MIR/NEG watershed based on the distribution pattern of the killifish *Austrolebias* in Uruguay. Similarly to *Cnesterodon*, *Austrolebias* inhabit shallow wetland habitats, and have little capabilities for active dispersal (Maltchik et al., 2014), reinforcing the relevance of passive geodispersal in the Pampas. In contrast, the most parsimonious explanation for the presence of haplotypes H21 and H30 in SOU is incomplete lineage sorting, given the low phylogenetic signal associated with location and clade structure for these haplotypes, and because the sampling points in which these haplotypes were found are distant from the URU watershed.

Our results suggest that the MRCA of *C. decemmaculatus* reached the lower Uruguay before 0.6 myr (**Figure 4**). The mtDNA genealogy suggests that from URU, *C. decemmaculatus* would have colonized NEG and SOU basins independently (**Figure 3**). The high genetic diversity in URU is consistent with its role as the ancestral location. Even though we have found a case of headwater capture from NEG to MIR, the most likely colonization route leading to MIR is through SOU, given that most haplotypes found in SOU and MIR belong to the same mtDNA haplogroup. The Bayesian delimitation of genetic populations also suggested that most localities in MIR and SOU belong to the same genetic population, which is also in agreement with pairwise indices of genetic structure (**Table 2**). These finding also highlight that, for this species, the genetic diversity in the lower Uruguay ecoregion would be much higher than in Laguna dos Patos (*sensu* Abell et al., 2008). Furthermore, given that URU is neighbor to SOU, but not MIR, a colonization route from URU to MIR and then SOU is less likely. Indeed, Bruno et al. (2016) suggested that the Argentinean coastal populations of *C. decemmaculatus* descend from upstream URU populations associated with the Rio de la Plata mouth, which highlight the mouth of the Uruguay river as a putative source for populations currently inhabiting isolated drainages flowing to Río de la Plata. Alternatively, SOU/MIR could have been colonized from NEG. This hypothesis would be strengthened if we assume that H10 evolved *in situ* in NEG before dispersing eastward. Additional sampling efforts, especially in NEG, but also in regions not sampled by this study, such as the Argentinian coast, will be required to refine these scenarios, as well as explicitly testing for hypotheses of stream capture or incomplete lineage sorting.

While the Pampean region has been always dominated by grasslands during the whole Pleistocene (Behling et al., 2005), changes in precipitation regimes and in the sea-level may have affected populations of *C. decemmaculatus*. For example, it could be expected that periods of increased humidity would have favored the formation of new wetland areas and promoted population expansion (Zemlak et al., 2008; Jones and Johnson, 2009). On the other hand, marine transgressions would have caused local extinctions (Villwock and Tomazelli, 1995). In this regard, the high genetic diversity in SOU was surprising, since this area suffered at least three marine transgression events since the Pleistocene (Villwock and Tomazelli, 1995) that could have led to declines in population size and local extinction. The high *N*_E_ and the significant negative values of Fu’s neutrality test for mtDNA indicate that either local extinction did not affect these populations, or that the high genetic diversity resulted from recent and strong population growth (**Table 3**). We also found evidence for population growth in MIR. However, differences in genetic diversity and *N*_E_ estimates between MIR and SOU may reflect different population histories after marine transgressions. Alternatively, some founder effect may have reduced genetic diversity in MIR following its colonization from SOU. Coastal populations from SOU may have also benefited from an extended coastal plain during marine retractions, facilitating population growth during these periods, as have been suggested for other species occurring along the coastal rivers of southern and southeastern Brazil (Thomaz et al., 2015).

## Author Contributions

AR-F, LM, and NF designed the study, interpreted the results, and contributed to the final version of the manuscript. AR-F did the laboratory work. AR-F and NF analyzed the data.

## Conflict of Interest Statement

The authors declare that the research was conducted in the absence of any commercial or financial relationships that could be construed as a potential conflict of interest.

## Footnotes

1 www.fluxus-engineering.com
