# Supplementary material for: Population Genetic Structure of Cnesterodon decemmaculatus (Poeciliidae): A Freshwater Look at the Pampa Biome in Southern South America
Source: Front Genet. 2017 Dec 19;8:214. doi: 10.3389/fgene.2017.00214 (PMC5742129; doi:10.3389/fgene.2017.00214)
Supplement: Supplementary file 1 [file Table_1.DOCX]

**Table S1.** Voucher specimens, drainage and broad drainage systems for samples used in this study. All specimens belong to the zoological fish collection at the Federal University of Rio Grande do Sul (UFRGS).

| **Voucher (each letter correspond to an individual)** | **Drainage/Population** | **Latitude** | **Longitude** |
| --- | --- | --- | --- |
| UFRGS 14561/TEC 0425 A, B, C, D, E | Uruguay/URU 1 | -32.2069 | -57.2130 |
| UFRGS 13811/TEC 0087 A, B, C, D, E | Uruguay/URU 2 | -32.0527 | -57.6719 |
| UFRGS 14566/TEC 0436 A, B | Uruguay/URU 3 | -31.7346 | -57.8669 |
| UFRGS 13812/TEC 0095 A, C, D, E | Uruguay/URU 4 | -31.6222 | -57.8808 |
| UFRGS 14549/TEC 0395 A, B, E | Uruguay/URU 5 | -31.4761 | -57.9016 |
| UFRGS 14538/TEC 0367 A, B, C, D, E | Uruguay/URU 6 | -31.3858 | -57.5627 |
| UFRGS 12372/TEC 0583 G, M, R, S, U | Uruguay/URU 7 | -31.3225 | -57.2825 |
| UFRGS 14550/TEC 0396 A, C, D, E, F | Uruguay/URU 8 | -31.2755 | -57.1561 |
| UFRGS 17859/TEC 3537 | Uruguay/URU 9 | -31.0354 | -56.8972 |
| UFRGS 14571/TEC 0456 A, B, G, H, I | Uruguay/URU 10 | -30.6586 | -56.6741 |
| UFRGS 14590/TEC 0496 A | Uruguay/URU 11 | -30.6308 | -57.6883 |
| UFRGS 13774/TEC 0001 | Uruguay/URU 11 | -30.6308 | -57.6883 |
| UFRGS 13912/TEC 0138 A, D, F | Uruguay/URU 12 | -30.5319 | -57.6658 |
| UFRGS 14640/TEC 0601 A, C, G, H, M, O | Uruguay/URU 13 | -30.4688 | -57.5122 |
| UFRGS 14552/TEC 0406 A | Uruguay/URU 14 | -30.4377 | -57.2961 |
| UFRGS 13783/TEC 0022 A, B, D, E, F | Uruguay/URU 15 | -29.8597 | -56.9369 |
| UFRGS 13784/TEC 0023 B, C, D, E | Uruguay/URU 16 | -30.2119 | -55.0550 |
| UFRGS 17874/ TEC 3552 | La Plata River/URU 17 | -33.9394 | -58.3663 |
| UFRGS 13927/TEC 0175 A, B | Negro River/NEG 35 | -33.4152 | -56.2016 |
| UFRGS 14637/TEC 0593 B | Negro River/NEG 36 | -31.9758 | -55.4702 |
| UFRGS 13933/TEC 0184 A, B, C, D | Negro River/NEG 37 | -31.9083 | -56.0177 |
| UFRGS 14531/TEC 0328 A, B, C, D | Negro River/NEG 38 | -31.3905 | -55.2538 |
| UFRGS 17852/TEC 3530 B | Mirim Lagoon/MIR 27 | -33.8295 | -54.7662 |
| UFRGS 17863/TEC 3541 A, B | Mirim Lagoon/MIR 28 | -33.5734 | -54.4987 |
| UFRGS 17873/TEC 3551 B | Mirim Lagoon/MIR 29 | -33.6136 | -54.3295 |
| UFRGS 18003/TEC 3675 A, B, C, D | Patos/MIR 30 | -33.4996 | -53.4310 |
| UFRGS 14576/TEC 0466 A, B, C, D | Mirim Lagoon/MIR 31 | -32.3661 | -54.1997 |
| UFRGS 17861/TEC 3539 | Mirim Lagoon/MIR 32 | -32.5190 | -53.4698 |
| UFRGS 17842/TEC 3520 A, B, C, D | Patos/MIR 34 | -31.9497 | -51.9600 |
| UFRGS 17869/TEC 3547 | Santa Lucia River/SOU 18 | -34.0118 | -56.9440 |
| UFRGS 17854/TEC 3532 A | La Plata River/SOU 19 | -34.4228 | -57.0112 |
| UFRGS 17866/TEC 3544 A, B | Santa Lucia River/SOU 20 | -34.5349 | -56.5765 |
| UFRGS 17865/TEC 3543 | Santa Lucia River/SOU 21 | -34.0989 | -56.2032 |
| UFRGS 17856/TEC 3534 | Santa Lucia River/SOU 22 | -34.2812 | -55.2784 |
| UFRGS 17870/TEC 3548 A, B | La Plata River/SOU 23 | -34.8415 | -55.0960 |
| UFRGS 17843/TEC 3521 | Rocha Lagoon/SOU 24 | -34.5133 | -54.2956 |
| UFRGS 17845/TEC 3523 | Atlantic Ocean Drainage/SOU 25 | -34.2061 | -53.7766 |
| UFRGS 17846/TEC 3524 | Atlantic Ocean Drainage/SOU 26 | -33.9204 | -53.5420 |
